# Longitudinal Visual Recovery and Exploratory Neuroimaging Findings in Pediatric Deprivation Amblyopia Following Unilateral Congenital Cataract Extraction: A Prospective Cohort Study

**DOI:** 10.3390/jcm15197696

**Published:** 2026-10-04

**Authors:** Shuhua Ni, Xiaoli Tang, Meng Li, Jing Li, Fei Leng, Panpan Zheng, Wei Shi, Li Li

**Affiliations:** 1 Key Laboratory of Major Diseases in Children, Ministry of Education, Department of Ophthalmology, Beijing Children’s Hospital, Capital Medical University, National Center for Children’s Health, Beijing 100045, China; 2 Department of Pulmonary and Critical Care Medicine, Beijing Tsinghua Changgung Hospital, School of Clinical Medicine, Tsinghua Medicine, Tsinghua University, Beijing 102218, China; 3 Department of Psychiatry and Psychotherapy, Jena University Hospital, Philosophenweg 3, 07743 Jena, Germany; 4 Department of Radiology, Beijing Tsinghua Changgung Hospital, School of Clinical Medicine, Tsinghua Medicine, Tsinghua University, Beijing 102218, China

**Keywords:** unilateral congenital cataract, deprivation amblyopia, central visual field, visual rehabilitation, magnetic resonance imaging

## Abstract

**Objectives**: Visual outcomes after unilateral congenital cataract (UCC) extraction remain variable, and the neuroanatomical changes accompanying subsequent visual improvement are poorly understood. This study characterized longitudinal changes in visual function and explored hemispheric asymmetry, longitudinal neuroimaging changes, and their associations with visual recovery in children after UCC extraction. **Methods**: This prospective observational cohort study enrolled 55 children aged 5 to <9 years with unilateral deprivation amblyopia receiving optical correction, passive occlusion, and dichoptic training. We analyzed 6-month changes in central visual field (CVF; mean sensitivity [MS], mean defect [MD]) and corrected distance visual acuity (CDVA). To assess structural alterations along the posterior visual pathways, a strictly controlled, exploratory subgroup of 18 right-handed patients (aged 5 to <8 years) with left-eye amblyopia underwent 3-Tesla (3T) MRI. Multiple comparisons in the neuroimaging and structure–function analyses were controlled using the Benjamini–Hochberg false discovery rate (FDR) procedure. **Results**: At 6 months, CDVA and CVF improved significantly from baseline across all amblyopia severity groups (all *p* < 0.05). Greater CDVA improvement was associated with larger increases in MS (β = 3.36, 95% CI, 1.37 to 5.35; *p* = 0.001) and greater reductions in MD (β = −2.80, 95% CI, −4.82 to −0.79; *p* = 0.007). Exploratory longitudinal neuroimaging analyses showed decreases in ipsilateral secondary visual cortex (V2) volume (Cohen’s d = −0.75, P_FDR_ = 0.042) and cortical thickness (Cohen’s d = −0.77, P_FDR_ = 0.042). Interhemispheric differences in V2 and middle temporal visual area (MT) cortical thickness were also observed at the 6-month follow-up (all |Cohen’s d| > 0.75, P_FDR_ < 0.05). Nominal structure–function associations were observed for CVF and CDVA changes (all |r| > 0.54, all *p* < 0.05). No serious adverse events were observed. **Conclusions:** Children with unilateral deprivation amblyopia following UCC extraction showed improvements in CDVA and CVF over 6 months of routine care. CVF perimetry may provide complementary functional information during longitudinal assessment. Exploratory neuroimaging findings suggested potentially distinct structural correlates of CDVA and CVF recovery, requiring validation in larger controlled longitudinal studies to clarify the clinical significance of CVF and its associated neuroimaging changes.

## 1. Introduction

Unilateral congenital cataract (UCC) is an important cause of severe deprivation amblyopia in children [1,2]. Even after early cataract extraction and appropriate postoperative management, including optical correction and occlusion therapy, visual outcomes remain highly variable [3,4]. In the Infant Aphakia Treatment Study (IATS), only 25% of treated children achieved a corrected distance visual acuity (CDVA) of 20/40 or better, whereas 44% had a CDVA of 20/200 or worse [4]. These findings illustrate the persistent challenge of visual recovery after early unilateral visual deprivation.

Persistent visual deficits associated with UCC are increasingly recognized as reflecting neurodevelopmental alterations beyond the eye itself. During early visual development, prolonged asymmetric visual input may alter activity-dependent neural maturation and trigger interocular competition within the posterior visual pathways [5]. Previous neuroimaging studies in children with deprivation amblyopia have reported abnormalities involving white matter (WM) integrity, optic radiations (ORs), primary visual cortex (V1), and lateral geniculate nucleus (LGN) [5,6,7,8,9,10]. These observations show that deprivation amblyopia is not limited to impaired foveal acuity but may involve a distributed neurodevelopmental disturbance across multiple levels of the visual system. Given the marked interocular suppression observed in children with deprivation amblyopia secondary to childhood cataract, binocular approaches such as dichoptic training have increasingly been used alongside occlusion therapy to rebalance binocular input, partially reduce suppression, and facilitate visual recovery [11,12,13,14].

CDVA remains the principal clinical endpoint for amblyopia, but it primarily reflects foveal spatial resolution and may not fully capture the broader functional consequences of early visual deprivation [15]. Automated perimetry in amblyopia provides a complementary assessment by characterizing the spatial distribution of visual sensitivity across the central visual field (CVF). Previous studies have demonstrated reduced visual-field sensitivity extending beyond the foveal region in amblyopic eyes, including generalized sensitivity depression across different amblyopia subtypes, such as deprivation amblyopia [16]. Consequently, CVF assessment may provide functional information that is not fully captured by visual acuity alone [17]. However, the longitudinal changes in CVF recovery after UCC extraction remain poorly characterized. It is also unclear whether CDVA and CVF follow similar temporal patterns during recovery. Furthermore, the neuroanatomical correlates of this functional recovery are poorly understood [5,6].

Therefore, in this prospective cohort study, we characterized 6-month changes in CVF and CDVA in children with UCC receiving combined routine care. We also examined the temporal patterns and clinical associations of these visual outcomes. In an exploratory MRI subgroup, we assessed hemispheric asymmetry and longitudinal structural changes within the posterior visual pathway and examined their associations with observed visual recovery. By combining multidimensional visual assessments with exploratory neuroimaging, we aimed to provide preliminary insights into the clinical and neuroanatomical features accompanying visual recovery after restoration of visual input in children with UCC.

## 2. Materials and Methods

### 2.1. Study Design

This single-center, prospective, observational study was conducted in adherence to the Declaration of Helsinki and was approved by the Institutional Review Board of Beijing Children’s Hospital on 8 April 2025 (reference No. 2025-Y-084-D).

### 2.2. Participants

Written informed consent was obtained from parents or legal guardians, alongside documented assent from children aged ≥ 8 years. Participants were prospectively enrolled beginning 10 April 2025, with the final participant completing their 6-month follow-up visit on 27 February 2026. A comprehensive summary of inclusion and exclusion criteria is summarized in Table S1.

We enrolled children aged 5 to <9 years with a history of unilateral dense central cataract extraction. Eligible participants required stable BCVA with optimal refractive correction, defined as wearing the same prescription for ≥16 weeks or exhibiting ≤1 logMAR line change between two measurements 8 weeks apart. Inclusion criteria comprised an amblyopic eye CDVA ranging from 0.2 to 1.0 logMAR (20/32 to 20/200), an age-appropriate fellow-eye CDVA of ≥20/32, an interocular CDVA difference of ≥2 logMAR lines, and an intact fellow eye CVF (mean sensitivity [MS] ≥25.0 decibel [dB], mean defect [MD] within ±2.0 dB). During follow-up, participants who did not adhere to the prescribed routine clinical management or developed ocular complications that interfered with visual training or subsequent outcome assessment were not included in the corresponding follow-up analysis [18,19].

### 2.3. Procedures and Clinical Outcomes

Clinical outcomes were evaluated at baseline, 3, and 6 months, including CDVA (Snellen, converted to logMAR), corrected near visual acuity (CNVA, Jaeger, Xingkang Medical Technology Co., Ltd., Wenzhou, China), CVF and near stereoacuity (Randot Preschool test; nil recorded as 10,000 arcseconds) [20]. For longitudinal analyses of monocular outcomes, including CDVA, CNVA, and CVF, only the amblyopic eye was analyzed at each time point. Central 10° CVF was measured using an Octopus 900 perimeter (Haag-Streit Diagnostics, Köniz, Switzerland) with the Macula program in EyeSuite software (v3.6.1) and the Tendency Oriented Perimetry strategy under white-on-white conditions. A Goldmann size III stimulus was presented for 100 ms against a 31.4-asb background. Tests with ≥15% false-positive/negative rates or ≥20% fixation losses were excluded. Quantitative CVF parameters were averaged from two consecutive reliable tests.

Following baseline evaluations, all enrolled subjects wore their optimal refractive correction full-time. In accordance with the Amblyopia Treatment Study guidelines, fellow-eye patching was prescribed according to amblyopia severity [21]. For severity classification, mild amblyopia was defined as an amblyopic-eye CDVA of 0.2–0.3 logMAR (approximately 20/30–20/40), moderate amblyopia as 0.4–0.6 logMAR (approximately 20/50–20/80), and severe amblyopia as 0.7–1.0 logMAR (approximately 20/100–20/200). Children with mild or moderate amblyopia were prescribed 2 h/day of patching, whereas those with severe amblyopia were prescribed 6 h/day. Dichoptic training was performed concurrently on a daily basis. Red–blue anaglyphic spectacles were used to present separate stimuli to the amblyopic and fellow eyes. Training was conducted on an 11.6-inch display at a viewing distance of approximately 60 cm. The amblyopic-eye stimulus flickered at 2 Hz. Fellow-eye contrast was adjusted individually between 20% and progressively increased according to training performance. Greater suppression was paired with lower fellow-eye contrast. Adherence was monitored using software logs and compliance scales, and treatment satisfaction was assessed using the Net Promoter Score [22].

### 2.4. Neuroimaging Acquisition and Processing

To maximize the signal-to-noise ratio, an exploratory homogeneous subcohort of 18 right-handed children aged 5 to <8 years with left-eye amblyopia underwent MRI at baseline and 6 months. All participants received oral chloral hydrate sedation at 50 mg/kg in accordance with institutional pediatric sedation procedures, with routine clinical observation before, during, and after MRI. Notably, the previous study indicates that chloral hydrate does not affect structural MRI [23]. Imaging was performed on a 3-Tesla MRI scanner (Ingenia CX; Philips, Medical Systems Nederland B.V., Best, The Netherlands) equipped with a 32-channel phased-array head coil. High-resolution three-dimensional (3D) T1-weighted images were acquired with a repetition time/echo time (TR/TE) of 8.18/3.799 ms, a flip angle of 8°, a field of view (FOV) of 240 × 240 mm^2^, and an acquisition matrix of 240 × 240. A total of 160 contiguous 1 mm slices were obtained, yielding an isotropic voxel resolution of 1 × 1 × 1 mm^3^. Diffusion tensor imaging (DTI) was acquired with a TR/TE of 8000/98.1 ms, a flip angle of 90°, an FOV of 240 × 240 mm^2^, and an acquisition matrix of 128 × 128. Diffusion weighting was applied at b = 1000 s/mm^2^ along 64 diffusion-encoding directions, including one b = 0 s/mm^2^ baseline image. The DTI spatial resolution was 2 × 2 × 2 mm^3^.

Before image processing, all structural images were visually inspected for motion artifacts and overall image quality. Bilateral lateral geniculate nucleus (LGN) volumes were segmented in native space using the Thalamus Optimized Multi-Atlas Segmentation pipeline [24]. Cytoarchitectonic regions of interest (ROIs), including V1, secondary visual cortex (V2), and the middle temporal area (MT), were defined using the BA_exvivo probabilistic atlas derived from histological data. Cortical thickness, surface area, and gray matter (GM) volume were extracted using FreeSurfer (v7.3.2). All cortical reconstruction and ROI parcellation outputs were visually reviewed for segmentation quality and anatomical plausibility before statistical analysis.

DTI data were preprocessed using FSL (v6.0.7.23) with correction for head motion and eddy-current distortions. Fiber orientation distributions (FODs) were then estimated via constrained spherical deconvolution in MRtrix3 (v3.0.8). Using the convolutional neural network-based TractSeg framework, 50 major WM tracts were automatically segmented from the FOD peak images. Tract orientation maps were converted into tract-specific tractograms for 3D streamline reconstruction. The bilateral ORs were selected as the primary tract ROIs, and reconstructed tracts were visually inspected for anatomical plausibility before tractometry. Quantitative tractometry was subsequently performed by dividing each reconstructed tract into 100 equal nodes to extract regional profiles of fractional anisotropy (FA) and apparent diffusion coefficient (ADC). To account for interindividual variability in global WM microstructure, mean FA and ADC values across all 50 segmented tracts were calculated and included as covariates in subsequent statistical models.

### 2.5. Outcomes

The primary correlation analysis evaluated the relationship between CVF changes and CDVA improvements from baseline to the 6-month follow-up. Additional exploratory analyses examined hemispheric asymmetry, longitudinal neuroanatomical changes, and structure–function associations between changes in visual function and structural measures, to further characterize the neuroanatomical correlates of visual recovery. Safety was assessed descriptively by recording adverse events (AEs) occurring during routine clinical care and follow-up [21,25].

### 2.6. Statistical Analysis

For the primary clinical evaluation, a pragmatic enrollment target of 55 participants was established with consideration of the planned multivariable regression model, which included five prespecified predictor parameters (CDVA improvement, two parameters representing amblyopia severity, central lens opacity diameter, and postoperative time), as well as expected attrition [26]. To minimize anatomical heterogeneity, the exploratory neuroimaging subcohort targeted 18 children (aged 5 to <8 years) with left-eye amblyopia. Analyses were conducted using R software (v4.5.0), with a two-sided significance level set at α = 0.05.

Test–retest reliability and variability of CVF parameters were assessed using intraclass correlation coefficients (ICCs) with 95% bootstrapped confidence intervals (CIs), non-parametric Bland–Altman analysis (median bias, 95% limits of agreement [LoA]), and coefficients of repeatability (CR) [27,28].

To evaluate longitudinal changes in clinical outcomes, generalized estimating equations (GEE) were used to assess the main effects of time, amblyopia severity, and their interaction. Intermittent 3-month outcome data missing in two participants who completed the 6-month follow-up were handled using multiple imputation before model fitting. Bonferroni adjustment was applied to post hoc comparisons. Paired *t*-tests or Wilcoxon signed-rank tests, as appropriate, were used to assess changes in posterior visual pathway measures from baseline to the 6-month follow-up. To account for multiple comparisons across neuroimaging measures, *p* values were adjusted using the Benjamini–Hochberg false discovery rate (FDR) procedure, with an FDR-adjusted *p* value (P_FDR_) < 0.05 considered statistically significant.

Stepwise multivariable linear regression analysis was used to assess associations between dichotomized BCVA improvements and changes in CVF parameters. BCVA improvement was dichotomized as 1 for an improvement of ≥1 logMAR/Jaeger line and 0 for an improvement of <1 logMAR/Jaeger line. Changes in MS and MD were calculated as the follow-up value minus the baseline value. Accordingly, a positive change in MS indicated increased CVF sensitivity, whereas a negative change in MD indicated a reduction in CVF defect and thus functional improvement. The regression model evaluating MS and CDVA was adjusted for postoperative time, central lens opacity diameter, and amblyopia severity. For post hoc pairwise comparisons among the three amblyopia severity levels, *p* values were adjusted using the FDR method to account for multiple testing. The model evaluating MS and CNVA was adjusted for central lens opacity diameter, while relationships between MD and visual acuity measures were assessed using univariable linear regression. Model performance was summarized using R^2^ and adjusted R^2^, together with the overall model *p* value. Regression assumptions were assessed via residual Q–Q plots and residual-versus-fitted plots. Homoscedasticity was formally assessed using the Breusch–Pagan test. For models with a Breusch–Pagan *p* value < 0.05, HC3 heteroscedasticity-consistent robust standard errors were used in sensitivity analyses. Potentially influential observations were assessed using Cook’s distance. Multicollinearity was evaluated using variance inflation factors (VIFs) in models containing multiple independent variables.

Within the neuroimaging subgroup, partial correlation analyses were performed to examine associations between longitudinal changes in neuroimaging measures and changes in visual function. Changes in CDVA, MS, MD and neuroimaging variables were calculated as the follow-up value minus the baseline value. The choice between Pearson and Spearman partial correlations was dictated by data distribution, which was assessed using the Shapiro–Wilk test. These analyses were rigorously adjusted for sex and the corresponding global neuroimaging change: regional volume changes were adjusted for changes in total intracranial volume (TIV); regional cortical thickness changes for changes in global mean cortical thickness; and OR indices (FA and ADC) for the corresponding global WM changes. Partial correlation coefficients and 95% CIs were calculated using Fisher’s Z-transformation for Pearson correlations and bootstrap resampling for Spearman correlations. *p* values for structure–function correlations were FDR-adjusted using the Benjamini–Hochberg procedure.

## 3. Results

Between 10 April and 27 August, 2025, 55 participants with unilateral amblyopia following extraction of a dense central cataract were enrolled. Based on baseline CDVA, participants were categorized into three groups: mild (*n* = 19), moderate (*n* =18), and severe amblyopia (*n* = 18). Fifty-three of the 55 participants (96.4%) had available 6-month outcome data and were included in the primary analysis (Figure S1). Of the two participants who did not complete follow-up, one developed posterior capsule opacification that affected subsequent visual training and outcome assessment, whereas the other did not return for the scheduled follow-up visit and was considered lost to follow-up.

The mean baseline age was 6.56 ± 1.18 years, with 56.4% females. Prior patching history, postoperative time, duration of refractive correction, and duration of opacity were comparable across severity groups (all *p* > 0.05; Table S2). However, central lens opacity diameter was significantly larger in the severe amblyopia group compared with the mild-to-moderate group (*p* = 1.83 × 10^−8^; Table S2).

In the neuroimaging subcohort (mean age, 6.28 ± 1.02 years), baseline TIV, total WM volume, and total GM volume were 1372.21 ± 90.02 cm^3^, 404.59 ± 38.54 cm^3^, and 767.84 ± 52.71 cm^3^, respectively. Regionally, the contralateral (right) V1 showed greater volume and thickness than the ipsilateral V1 (all P_FDR_ < 0.05; Table 1). Conversely, the left OR demonstrated a higher ADC than the right OR (P_FDR_ = 0.006; Table 1). In addition, right V2 showed nominally greater cortical thickness than left V2, although this difference did not remain significant after FDR correction (*p* = 0.045, P_FDR_ = 0.101; Table 1). Of the 18 participants in the neuroimaging subcohort, 17 (94.4%) completed the 6-month MRI assessment and were included in the longitudinal imaging analysis (Figure S1).

### 3.1. Subsection

#### 3.1.1. Longitudinal Changes in Visual Function and Structural Neuroanatomy

**Figure 1:**
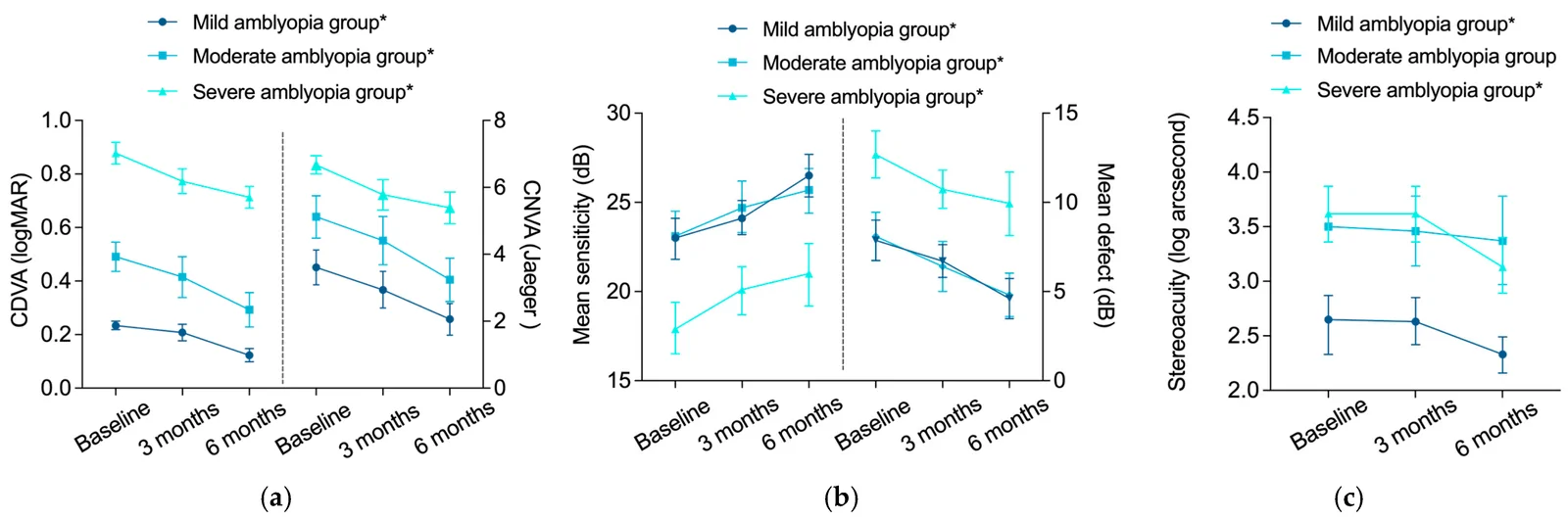
Longitudinal changes in visual function parameters during the 6-month follow-up. Trajectories of (**a**) best-corrected visual acuity, (**b**) central visual field, and (**c**) stereoacuity, analyzed using generalized estimating equation models. * Denotes statistically significant differences between baseline and the 6-month endpoint within the mild, moderate, and severe amblyopia groups. None of the time-by-group interactions were statistically significant; CDVA, corrected distance visual acuity; CNVA, corrected near visual acuity; dB, decibel.

At the 6-month follow-up, 90.57% of participants showed an improvement of ≥1 logMAR line from baseline. CDVA and CVF improved significantly over 6 months across all amblyopia severity groups, with no significant time-by-severity interactions (Figure 1). Interval analyses suggested descriptive differences in the timing of CDVA improvement across severity groups (Table S3). The severe group showed greater improvement during the first 3 months, followed by less change between 3 and 6 months, whereas the mild and moderate groups showed more apparent improvement during the latter interval. Stereoacuity improvement was observed mainly between 3 and 6 months. A significant late-phase improvement was observed in the severe group (*p* = 0.029), whereas the mild group showed a nonsignificant trend toward improvement (*p* = 0.078) and the moderate group showed no detectable change (*p* = 1.000).

**Figure 2:**
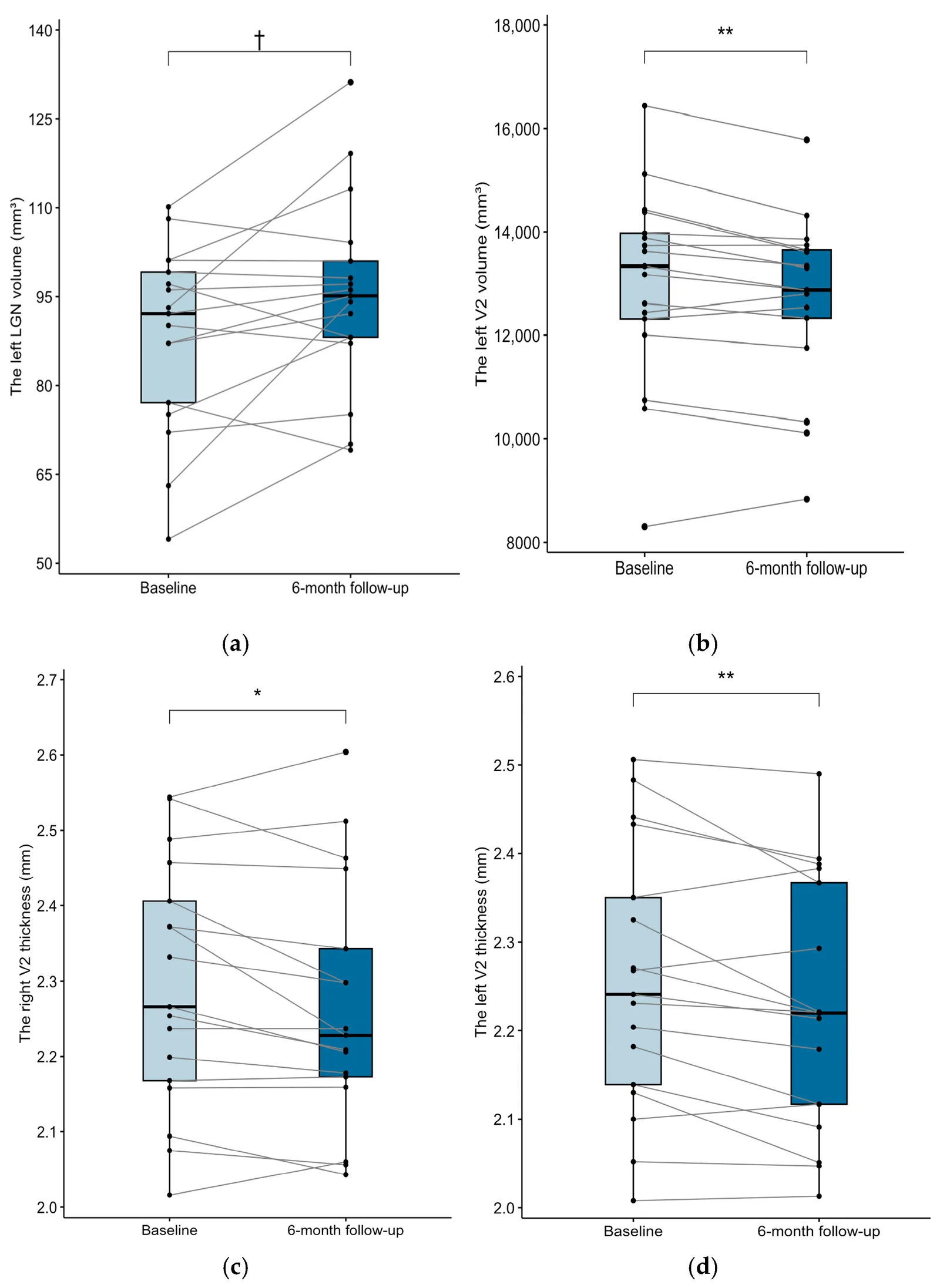
Longitudinal structural alterations in the posterior visual pathways within the neuroimaging subcohort. Spaghetti plots of (**a**) ipsilateral lateral geniculate nucleus (LGN) volume, (**b**) ipsilateral secondary visual cortex (V2) volume, (**c**) contralateral V2 cortical thickness, and (**d**) ipsilateral V2 cortical thickness, analyzed using paired *t*-tests, with group means and 95% CIs overlaid. ^†^ indicates a nominal association at an unadjusted *p* < 0.05; asterisks indicate statistically significant differences between baseline and the 6-month post-treatment endpoint (* *p* < 0.05, ** *p* < 0.01).

In the exploratory neuroimaging subset, TIV and total WM volume increased over 6 months (Cohen’s d = 0.89, P_FDR_ = 0.013 and Cohen’s d = 1.22, P_FDR_ = 0.003, respectively), whereas the remaining global structural measures showed no nominal longitudinal changes (all |Cohen’s d| < 0.3, *p* > 0.05; Table S4). At 6 months, exploratory interhemispheric comparisons showed greater right V1 volume and cortical thickness and greater right V2 cortical thickness than on the left (all P_FDR_ < 0.05; Table S5). The left OR showed higher ADC values than the right OR, and left MT cortical thickness was greater than that on the right (both P_FDR_ < 0.05; Table S5). Longitudinal regional analyses showed a nominal increase in ipsilateral LGN volume (Cohen’s d = 0.58, *p* = 0.029, P_FDR_ = 0.139), but ipsilateral V2 volume decreased significantly after FDR correction (Cohen’s d = −0.75, *p* = 0.007, P_FDR_ = 0.042). V2 cortical thickness decreased nominally in the contralateral hemisphere (Cohen’s d = −0.53, *p* = 0.046, P_FDR_ = 0.183) and significantly in the ipsilateral hemisphere (Cohen’s d = −0.77, *p* = 0.006, P_FDR_ = 0.042). More than 65% of participants showed changes in the same direction for these three ROI measures (Figure 2).

#### 3.1.2. Primary Correlative Analyses in the Full Cohort

- Multivariable associations with mean sensitivity improvement

**Figure 3:**
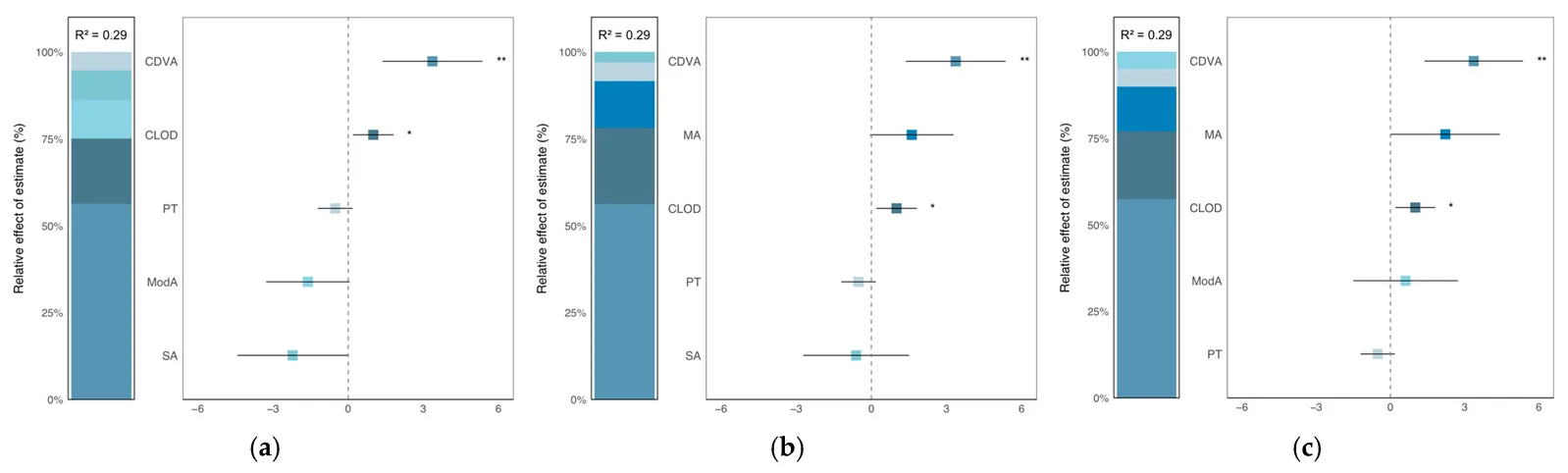
Multiple linear regression models for MS improvement. The panels illustrate the associations of PT, CLOD, CDVA, and amblyopia severity with MS improvement, using the (**a**) mild, (**b**) moderate, and (**c**) severe amblyopia groups as reference categories, respectively. Asterisk indicates statistical significance (* *p* < 0.05, ** *p* < 0.01); CDVA, improvement of corrected distance visual acuity; CLOD, central lens opacity diameter (mm); MA, mild amblyopia group; ModA, moderate amblyopia group; PT, postoperative time (years); SA, severe amblyopia group.

Multivariable analysis showed that greater CDVA improvement was associated with greater MS improvement (β = 3.36, 95% CI, 1.37 to 5.35; *p* = 0.001). The model explained 29.15% of the variance in MS improvement (adjusted R^2^ = 0.2161; *p* = 0.005). A larger central lens opacity diameter was also independently associated with greater MS improvement (β = 1.00, 95% CI, 0.19 to 1.82; *p* = 0.017), whereas postoperative time was not significantly associated with mean sensitivity improvement (β = −0.51, 95% CI, −1.21 to 0.18; *p* = 0.142). Differences among the mild, moderate, and severe amblyopia groups did not remain significant after FDR correction (all P_FDR_ > 0.05; Figure 3 and Table S6). The Breusch–Pagan test showed no evidence of heteroscedasticity (*p* = 0.261), and all Cook’s distances were <1 (Figure S2).

- In a separate model, greater CNVA improvement was associated with greater MS improvement after adjusting for central lens opacity diameter (β = 3.65, 95% CI, 0.94 to 6.37; *p* = 0.009). The model explained 15.09% of the variance in MS improvement (adjusted R^2^ = 0.1169; *p* = 0.017). Central lens opacity diameter was not significantly associated with MS improvement in this model (β = 0.46, 95% CI, −0.08 to 0.99; *p* = 0.099). The Breusch–Pagan test indicated heteroscedasticity (*p* = 0.008). In the HC3 sensitivity analysis, the association between CNVA improvement and MS improvement was β = 3.65 (95% CI, −1.62 to 8.93; *p* = 0.183), and the association between central lens opacity diameter and MS improvement was β = 0.46 (95% CI, −0.15 to 1.06; *p* = 0.147).
- Univariable associations with mean defect changes

MD changes were negatively associated with both CDVA improvement (β = −2.80, 95% CI, −4.82 to −0.79; *p* = 0.007; R^2^ = 0.1329, adjusted R^2^ = 0.1159) and CNVA improvement (β = −3.15, 95% CI, −5.78 to −0.53; *p* = 0.020; R^2^ = 0.1024, adjusted R^2^ = 0.0848). For the CDVA model, the Breusch–Pagan test showed no evidence of heteroscedasticity (*p* = 0.211), and all Cook’s distances were <1 (Figure S3). For the CNVA model, the Breusch–Pagan test indicated heteroscedasticity (*p* = 0.019); however, the association was no longer statistically significant in the HC3 robust standard error sensitivity analysis (β = −3.15, 95% CI, −8.50 to 2.20; *p* = 0.243).

#### 3.1.3. Exploratory Clinical and Neuroanatomical Correlates of Visual Recovery in the MRI Subgroup

- Neuroimaging Correlates of CDVA changes

**Figure 4:**
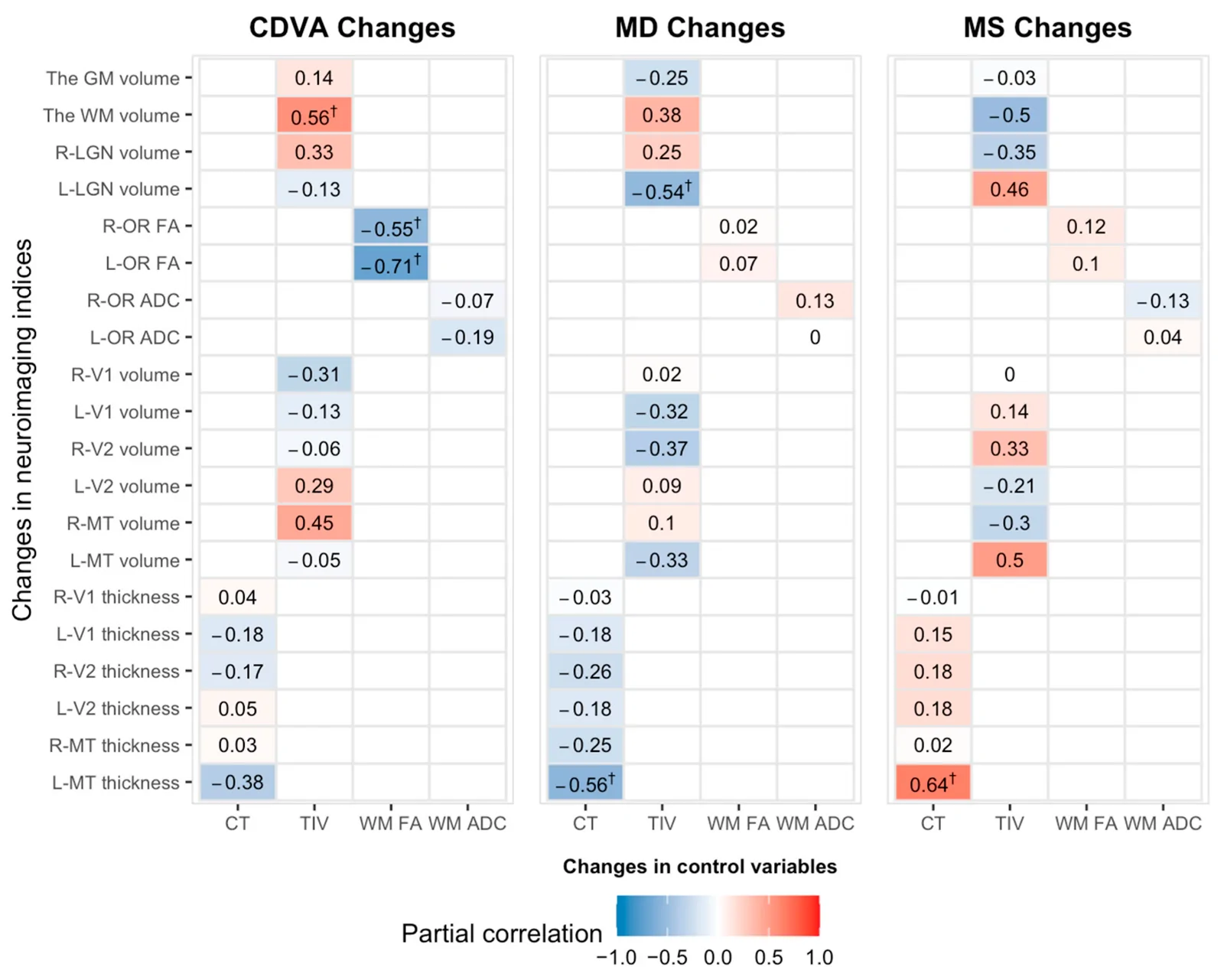
Heatmap illustrating the associations between changes in neuroimaging indices and clinical visual outcomes, adjusting for relevant covariates. The top panels display the corresponding changes in clinical outcomes. Color gradients reflect the correlation coefficients of these associations. ^†^ indicates a nominal association at an unadjusted *p* < 0.05; none of the associations remained statistically significant after false discovery rate correction. ADC, apparent diffusion coefficient; CDVA, corrected distance visual acuity; FA, fractional anisotropy; L, left; LGN, lateral geniculate nucleus; MCT, mean cortical thickness; MD, mean defect; MS, mean sensitivity; MT, middle temporal visual area; OR, optic radiation; R, right; TIV, total intracranial volume; V1, primary visual cortex; V2, secondary visual cortex; WM, white matter.

Changes in total WM volume showed a nominal positive association with changes in CDVA (r = 0.56, 95% CI, 0.07 to 0.83; *p* = 0.029; P_FDR_ = 0.358). FA changes in the bilateral ORs showed nominal inverse associations with changes in CDVA, including the ipsilateral OR (r = −0.71, 95% CI, −0.90 to −0.32; *p* = 0.003; P_FDR_ = 0.171) and contralateral OR (r = −0.55, 95% CI, −0.83 to −0.05; *p* = 0.035; P_FDR_ = 0.358). None of these associations remained statistically significant after FDR correction (Figure 4; Table S7a). Total GM volume and structural changes in the remaining ROIs were not associated with CDVA changes (Figure 4; Table S7a).

- Neuroimaging Correlates of CVF changes

Changes in ipsilateral MT cortical thickness showed nominal associations with changes in CVF measures. Greater increases in MT cortical thickness were associated with increases in MS (r = 0.64, 95% CI, 0.19 to 0.87; *p* = 0.010; P_FDR_ = 0.358) and decreases in MD (r = −0.56, 95% CI, −0.83 to −0.06; *p* = 0.031; P_FDR_ = 0.358). Changes in ipsilateral LGN volume were also nominally associated with changes in MD (r = −0.54, 95% CI, −0.83 to −0.04; *p* = 0.036; P_FDR_ = 0.358). None of these associations remained statistically significant after FDR correction, and other regional structural changes showed no evidence of association with CVF changes at the unadjusted level (Figure 4; Table S7b,c).

#### 3.1.4. Additional Outcomes

- Treatment Adherence and Participant Satisfaction

Mean compliance scores remained high at 3 (78.49) and 6 months (79.38) [18]. Patient-reported and system-logged completion rates were highly concordant at 3 (87.03% vs. 86.69%, *p* = 0.084) and 6 months (87.69% vs. 87.40%, *p* = 0.028). Furthermore, 85.19% of parents with prior patching experience preferred this therapy, yielding a 6-month NPS question of 66.04 (Table S8).

- CVF Reliability and Measurement Agreement

Averaging consecutive measurements significantly improved reliability over single tests, yielding exceptionally high ICCs for both MD (≥0.98) and MS (≥0.96) across all time points (Table S9a). Moreover, Bland–Altman analysis confirmed robust long-term stability with no systematic bias (Table S9b), corroborated by improvements in the CR from baseline to 6 months for both MD (1.92 to 1.73 dB) and MS (2.40 to 1.83 dB).

- Safety Outcomes

No serious AEs or theoretical dichoptic training complications were observed. Non-serious AEs occurred in 12.7% (7/55) of patients, comprising four unrelated systemic events involving infections or allergies, and three ocular events, specifically one posterior capsule opacification in moderate amblyopia and two new-onset heterotropias in severe amblyopia. No participant discontinued dichoptic training because of intolerance.

## 4. Discussion

The present study provides a longitudinal characterization of visual recovery in children with deprivation amblyopia following UCC extraction, complemented by exploratory neuroimaging analyses. By integrating longitudinal functional metrics with high-resolution structural MRI mapping, we observed temporal recovery dynamics, concurrent functional improvements, changes in interhemispheric patterns and suggestive distinct neuroimaging associations across visual recovery domains.

### 4.1. Best-Corrected Visual Acuity and Central Visual Field Recovery Profiles

Over the 6-month period, the mean improvement in CDVA of the amblyopic eye was 1.55 lines, with most participants achieving the predefined responder threshold. A previous study of patching alone in deprivation amblyopia reported a mean CDVA gain of 0.2 lines over a similar follow-up period [11]. While the greater improvement observed in our cohort is noteworthy, differences in study populations, treatment context, and study design preclude direct comparison. Accordingly, these findings should be interpreted as descriptive and hypothesis-generating rather than as evidence of an additive or synergistic treatment effect.

Although CDVA remains the standard metric for monitoring amblyopia recovery, it primarily measures high-contrast foveal resolution and may not fully capture broader spatial deficits [17,29,30]. This limitation may be particularly relevant in children with dense central cataracts, in whom visual deprivation can result in persistent amblyopia and may affect visual function beyond central acuity [31]. Previous studies have also reported persistent postoperative visual-field deficits after congenital cataract surgery [32]. In the present longitudinal cohort, CVF measures improved over 6 months during routine visual rehabilitation, supporting the potential value of CVF as a complementary measure of functional recovery beyond conventional acuity assessment. Pediatric perimetry is influenced by attention and test performance [33]. Averaging repeated reliable measurements may improve measurement stability, while the neuroanatomical basis of CVF recovery remains to be further investigated.

### 4.2. Temporal Patterns of Visual Recovery During Follow-Up

Longitudinal tracking showed descriptively different temporal patterns of visual improvement across baseline severity groups; however, the nonsignificant time-by-severity interaction does not support a definitive severity-dependent recovery pattern. The severe group showed greater CDVA improvement during the first 3 months, whereas the mild and moderate groups showed more apparent improvement between 3 and 6 months. These descriptive differences may partly reflect greater potential for measurable improvement among participants with poorer baseline vision [13,34]. They should therefore be interpreted cautiously and require confirmation in larger cohorts.

Stereoacuity showed a later pattern of improvement than CDVA and CVF, with changes becoming more apparent between 3 and 6 months. Previous studies have shown that stereoacuity and other binocular visual functions can improve during visual training in amblyopia [35,36,37]. In the present cohort, late-phase stereoacuity improvement reached statistical significance only in the severe group. The mild group showed a nonsignificant trend, whereas the moderate group showed no detectable change. These subgroup patterns may suggest differences in the timing of stereoacuity improvement across baseline severity levels. However, because the time-by-severity interaction was not statistically significant, these observations should be interpreted as descriptive rather than as evidence of distinct severity-specific recovery mechanisms. Larger controlled longitudinal studies are needed to determine whether stereoacuity recovery truly differs according to baseline amblyopia severity.

### 4.3. Clinical Correlates of Central Visual Field Improvement

Beyond the temporal patterns of visual recovery, greater CDVA improvement was associated with greater CVF improvement, reflected by larger increases in MS and greater reductions in MD. More extensive preoperative form deprivation was also associated with greater MS improvement in the multivariable model. Associations involving CNVA were less robust in sensitivity analyses. Previous automated perimetry studies have shown that amblyopia may be accompanied by generalized reductions in visual-field sensitivity, including abnormalities in foveal threshold and mean deviation [16]. In the present study, CDVA and CVF changes were related during follow-up, but the regression models explained only a modest proportion of the variance in CVF recovery. The complementary clinical value of CVF beyond conventional visual acuity measures therefore requires further evaluation in larger longitudinal studies.

### 4.4. Exploratory Neuroanatomical Findings

By restricting the MRI subcohort to children aged 5 to <8 years, we sought to reduce age-related developmental heterogeneity while retaining a developmental period in which visual plasticity remains active [38,39]. At baseline, rightward asymmetry was observed in V1 volume and cortical thickness, whereas ADC was higher in the left OR. Right V2 cortical thickness also showed a nominal interhemispheric difference that did not remain significant after FDR correction. Similar developmental asymmetries have been reported in typically developing children, including rightward cortical-thickness asymmetry in posterior occipital regions and left-lateralized diffusion metrics within the OR. These observations suggest that at least part of the hemispheric asymmetry identified in our cohort may reflect normal developmental lateralization rather than deprivation-specific reorganization [40,41]. Notably, the OR asymmetry was also broadly consistent with left-eye anisometropic amblyopia, while differing from patterns in left-eye strabismic amblyopia [42].

These interhemispheric patterns were also evident at the 6-month follow-up. Rightward V2 thickness became significant at 6 months, while leftward MT thickness emerged during follow-up. The MT finding differed from previous observations in anisometropic amblyopia and may relate to differences in amblyopia subtype or visual experience [43]. Longitudinally, TIV and WM volume increased, ipsilateral LGN volume showed a nominal increase, and ipsilateral V2 volume and thickness decreased significantly. Because subcortical structures, cortical thickness, and white-matter microstructure continue to undergo age-related maturation during childhood, these findings should be interpreted cautiously [44,45]. Future longitudinal studies incorporating age-matched healthy controls are needed to determine the extent to which these asymmetries and temporal changes reflect deprivation-related processes rather than normal developmental variation.

Recent neuroimaging studies in children with anisometropic amblyopia have demonstrated measurable structural and functional changes during visual recovery, but comparable longitudinal evidence in deprivation amblyopia remains limited [46]. As illustrated in Figure 4, CDVA change showed nominal associations with total WM volume and bilateral OR FA, whereas CVF change showed nominal associations with ipsilateral LGN volume and MT cortical thickness. None of these associations remained statistically significant after FDR correction. These exploratory patterns raise the possibility that different aspects of visual recovery may be associated with different structural changes within the posterior visual pathway. We also observed that longitudinal changes in V2 were not significantly associated with improvements in visual function, suggesting that V2 morphometric changes may primarily reflect developmental or experience-dependent processes [46]. Pending such validation, CVF may provide complementary functional information beyond standard CDVA, although the structural correlates of CVF recovery remain to be clarified [47].

Several limitations merit careful consideration. First, the absence of a randomized control cohort prevents attributing all functional and neuroanatomical alterations exclusively to dichoptic training. Multicentre randomized trials are therefore required to determine comparative efficacy. Second, while strict MRI criteria optimized the signal-to-noise ratio, the small neuroimaging sample size restricts statistical power and generalizability, rendering these findings preliminary. The absence of longitudinal age-matched healthy controls further prevents clear separation of deprivation-related changes from normal developmental trajectories. Third, the 6-month observation period captures only an early phase of recovery and does not establish whether the observed clinical and neuroimaging patterns are sustained over longer periods. Larger controlled longitudinal studies with repeated imaging and age-matched healthy controls are warranted to confirm these findings.

## 5. Conclusions

Children with unilateral deprivation amblyopia following UCC extraction showed improvements in CDVA and CVF over 6 months of routine visual rehabilitation. Exploratory MRI analyses showed longitudinal structural and interhemispheric changes within the posterior visual pathway, although their developmental and clinical significance remains uncertain. Structure–function associations were nominal and did not survive FDR correction. CVF perimetry may provide complementary functional information beyond conventional visual acuity assessment, but its clinical value requires further validation in larger controlled longitudinal studies.

## Figures and Tables

**Table 1:** Interhemispheric comparison of baseline morphometric and microstructural parameters in the posterior visual pathways.

Characteristics	Right Hemisphere (*n* = 18)	Left Hemisphere (*n* = 18)	Cohen’s d	*p* Value	P_FDR_ Value
The LGN volume (mm^3^)	95.85 ± 17.08	90.23 ± 16.75	0.39	0.119	0.179
Optic radiations					
FA	0.43 ± 0.02	0.43 ± 0.02	0.20	0.404	0.455
ADC (x 10-3 mm^2^/s) *	0.82 ± 0.05	0.86 ± 0.05	−0.87	0.002	0.006
Primary visual cortex					
Thickness (mm) *	1.86 ± 0.17	1.74 ± 0.12	0.95	0.001	0.004
Volume (mm^3^) *	4981.56 ± 962.36	4064.50 ± 703.56	1.71	1.326 × 10^−6^	1.193 × 10^−5^
Secondary visual cortex					
Thickness (mm) ^†^	2.28 ± 0.16	2.25 ± 0.15	0.51	0.045	0.101
Volume (mm^3^)	13,029.39 ± 1949.47	13,077.00 ± 1885.01	−0.06	0.815	0.815
Middle temporal visual area					
Thickness (mm)	2.68 ± 0.15	2.76 ± 0.12	−0.43	0.086	0.154
Volume (mm^3^)	4582.44 ± 818.65	4284.78 ± 566.79	0.35	0.150	0.193

Data are presented as mean ± SD, with statistical comparisons evaluated via a paired *t*-test. ^†^ indicates a nominal association at an unadjusted *p* < 0.05. * Denotes significant inter-group differences. ADC = apparent diffusion coefficient; FA = fractional anisotropy; FDR = false discovery rate; LGN = lateral geniculate nucleus; MRI = magnetic resonance imaging.

## Data Availability

The datasets generated and/or analyzed during the current study are not publicly available because the informed consent agreements permit only the publication of aggregated statistical data. Deidentified data may be made available by the corresponding authors upon reasonable request, subject to applicable ethical and privacy restrictions, to facilitate verification and reproducibility of the reported findings.

## Supplementary Materials

The following supporting information can be downloaded at: https://www.mdpi.com/article/10.3390/jcm15197696/s1, Figure S1: Flowchart showing trial profile; Figure S2: Residual diagnostics for the multivariable linear regression model of mean sensitivity improvement; Figure S3: Residual diagnostics for the univariable linear regression model of mean defect change with the improvement of corrected distance visual acuity as the independent variable; Table S1: Eligibility criteria; Table S2: Baseline demographic and ophthalmic characteristics; Table S3: Mean changes in visual function during the 0–3 and 3–6 month interval; Table S4: Longitudinal changes in neuroimaging characteristics in the MRI subcohort; Table S5: Interhemispheric comparison of posterior visual pathway morphometric and microstructural measures at the 6-month follow-up; Table S6: Factors associated with improvements in mean sensitivity and corrected distance visual acuity; Table S7: The associations between changes in neuroimaging indices and clinical visual outcomes; Table S8: Therapeutic patient satisfaction; Table S9: The repeatability, reliability, and variability of central visual field examinations at each visit.

## Abbreviations

The following abbreviations are used in this manuscript:

UCC	Unilateral congenital cataract
CVF	Central visual field
MS	Mean sensitivity
MD	Mean defect
CDVA	Corrected distance visual acuity
3T	3-Tesla
MRI	Magnetic resonance imaging
FDR	False discovery rate
IATS	Infant Aphakia Treatment Study
WM	White matter
V1	Primary visual cortex
LGN	Lateral geniculate nucleus
dB	Decibel
CNVA	Corrected near visual acuity
NPS	Net Promoter Score
DTI	Diffusion tensor imaging
V2	Secondary visual cortex
MT	Middle temporal visual area
GM	Gray matter
OR	Optic radiation
FA	Fractional anisotropy
ADC	Apparent diffusion coefficient
AE	Adverse event
ICC	Intraclass correlation coefficient
CI	Confidence interval
LoA	Limits of agreement
CR	Coefficient of repeatability
GEE	Generalized estimating equations
VIFs	Variance inflation factors
TIV	Total intracranial volume
