# Supplementary material for: Longitudinal Visual Recovery and Exploratory Neuroimaging Findings in Pediatric Deprivation Amblyopia Following Unilateral Congenital Cataract Extraction: A Prospective Cohort Study
Source: J Clin Med. 2026 Oct 4;15(19):7696. doi: 10.3390/jcm15197696 (PMC13666433; doi:10.3390/jcm15197696)
Supplement: Supplementary file 1 [file jcm-15-07696-s001.zip › Supplementary_tables.pdf]

Supplementary Table S1. Eligibility criteria

Inclusion criteria

Participants were eligible for enrollment if they satisfied the following criteria:

1. A signed and dated informed consent form
2. Age 5 to < 9 years, male and female
3. Unilateral amblyopia following the extraction of a dense central cataract
4. Strabismus limited to tropia of 5 PD or less measured via the SPCT at near fixation or heterophoria up to 10 PD measured via the APCT under best-corrected optical condition.
5. An intact CVF in the fellow eye, defined as a MS of 25.0 dB or greater and a MD within ± 2.0 dB.
6. Must have refractive error correction (based on a cycloplegic refraction completed within the previous 7 mos) if any of the following are true:
   a. Hypermetropia of 2.50 D or more by SE
   b. Myopia of the amblyopic eye of 0.50 D or more
   c. Astigmatism of 1.00 D or more
   d. Anisometropia of more than 0.50 D
7. Optical adaptation and CDVA stability requirements:

Duration: Documented adherence to spectacle correction for at least 16 weeks, or until CDVA stability is achieved

a. Definition of stability (nonimprovement): A difference of < 0.1 logMAR between two consecutive assessments performed at least 8 weeks apart using the same testing method

b. Initial measurement: Utilizing current spectacles, or trial frames with or without cycloplegia, or uncorrected vision (if a new correction is prescribed)

c. Subsequent Measurement: Noncycloplegic reassessment with prescribed spectacles worn for at least 8 weeks

8. CDVA, measured in each eye without cycloplegia with current refractive correction (if applicable) within 7 days prior to treatment, as follows:
   a. CDVA in the amblyopic eye of 20/32 to 10/100
   b. CDVA in the fellow eye of 20/32 or better
   c. A minimum interocular CDVA difference of 2 logMAR lines
9. Patients in general good health and able, as per investigator determination, to comply with the study visits and protocol procedures, to wear refractive correction, and who have access to wireless internet service at home that is able to support the combined DT and occlusion treatment regimen.

Exclusion criteria

Individuals with any of the following characteristics were excluded from the study:

1. Prior DT treatment
2. A history of mental disorders or use of medications affecting the nervous system
3. Drug-induced cataract
4. Suspected microphthalmia (axial length ≤19.0 mm)
5. Myopia > -6.00D SE in either eye
6. Known skin reactions to patch or bandage adhesives
7. Active ocular and systemic diseases that could be a potential cause for reduced CDVA according to the investigators
8. Previous intraocular surgery other that cataract extraction and/or IOL implantation
9. CVF reports with false-positive rate > 15%, or false-negative rate > 20%, or fixation loss rate > 20%

10. Poor compliance:

a. a total score ≤ 70 points on a compliance assessment scale＊[18]

b. DT completion rate ≤ 80%, based on parent-reported or system-logged data[19]

11. Patients who do not wear their spectacles and adhere to scheduled patching regimens (as assessed by investigator)

12. A history of light-induced seizures

＊The 18-item postoperative visual function rehabilitation compliance scale for children with congenital cataract, which comprehensively assesses compliance across five dimensions (background factors, intrinsic motivation, cognitive appraisal, emotional response, and interaction)，with each item scored on a 5-point Likert scale anchored from 1 ("Completely inconsistent") to 5 ("Completely consistent").

APCT, alternate prism cover test; CDVA, corrected distance visual acuity; CVF, central visual field; D, diopter; dB, decibel; DT, dichoptic training; IOL, intraocular lens; logMAR, logarithm of the minimum angle of resolution; MS, mean sensitivity ; MD, mean defect; PD, prism diopters; PL, perceptual learning; SE, spherical equivalent; SPCT, simultaneous prism and cover test

Supplementary Table S2. Baseline demographic and ophthalmic characteristics

Characteristic	Mild amblyopia group (n =19)	Moderate amblyopia group (n = 18)	Severe amblyopia group (n = 18)	P value
Age (yrs)	6.67 ± 1.36	6.52 ± 1.17	6.54 ± 1.00	0.907
Sex				
Male	9/19 (47.4)	8/18 (44.4)	7/18 (38.9)	0.687
Female	10/19 (52.6)	10/18 (55.6)	11/18 (61.1)	0.687
Prior amblyopic treatment				
None	9/19 (47.4)	9/18 (50.0)	10/18 (55.6)	0.880
Patching	10/19 (52.6)	9/18 (50.0)	8/18 (44.4)	0.880
Atropine	0/19 (0.0)	0/18 (0.0)	0/18 (0.0)	1.000
Perspective learning	0/19 (0.0)	0/18 (0.0)	0/18 (0.0)	1.000
Prior strabimus surgery	0/19 (0.0)	0/18 (0.0)	0/18 (0.0)	1.000
Amblyopic eye				
Right eye	7/19 (36.8)	9/18 (50.0)	7/18 (38.9)	0.871
Left eye	12/19 (63.2)	9/18 (50.0)	11/18 (61.1)	0.871
Duration of current Rx (yrs)	3.80 ± 1.27	3.59 ± 1.41	4.42 ± 0.82	0.106
Lens opacity time (mos)	3.74 ± 1.90	4.02 ± 1.86	4.61 ± 1.76	0.102
Postoperative time (yrs)	4.00 ± 1.27	3.80 ± 1.39	4.63 ± 0.82	0.352
Lens opacity diameter (mm)*	3.04 ± 0.92☆	3.22 ± 1.16☆	5.09 ± 0.75◇△	1.83×10^{-8}
Baseline CDVA (logMAR)				
Amblyopic eye*	0.22 (0.20,0.24)△☆	0.46 (0.40,0.58)◇☆	0.86 (0.80,1.00)◇△	3.18×10^{-11}
Fellow eye	0.00 (0.00,0.05)	0.00 (0.00,0.04)	0.05 (0.00,0.09)	0.21
Baseline CNVA (Jaeger)				
Amblyopic eye*	4.00 (3.00,4.00)△☆	5.00 (4.00,6.00)◇☆	7.00 (6.25,7.00)◇△	8.92×10^{-8}
Fellow eye	1.00 (1.00,2.00)	1.00 (1.00,2.00)	1.00 (1.00,2.00)	0.89
Mean sensitivity (dB) in CVF				
Amblyopic eye*	22.99 ± 2.47☆	22.83 ± 3.07☆	17.94 ± 3.25◇△	1.74×10^{-6}
Fellow eye	30.23 ± 1.42	30.86 ± 0.87	30.32 ± 1.09	0.218
Mean defect (dB) in CVF				
Amblyopic eye*	7.86 ± 2.43☆	8.39 ± 3.11☆	12.68 ± 2.90◇△	3.80×10^{-6}
Fellow eye	0.75 ± 1.10	0.28 ± 0.94	0.50 ± 0.67	0.311
Stereoacuity (log arcsecond)*	2.60 (2.30,2.90)△☆	4.00 (2.90,4.00)◇	4.00 (2.90,4.00)◇	1.72×10^{-5}

Data were presented as no./total no. (%) for categorical variables, mean ± standard deviation for normally distributed quantitative variables with equal variances, and median (interquartile range) for those with non-normal distribution or unequal variances.

* Denotes significant inter-group differences; ◇ Denotes significant differences compared with the mild amblyopia group; △ Denotes significant differences compared with the moderate amblyopia group; ☆ Denotes significant differences compared with the severe amblyopia group.

CDVA, corrected distance visual acuity; CNVA, corrected near visual acuity; CVF, central visual fields; dB, decibel; logMAR, logarithm of the minimum angle of resolution; Rx, vision prescription

Supplementary Table S3. Mean changes in visual function during the 0-3 and 3-6 month interval

Characteristics		0-3 month follow-up				3-6 month follow-up			
		MD	SE	95% CI	P value	MD	SE	95% CI	P value
CDVA (logMAR)	Mild amblyopia	0.03	0.02	(-0.02,0.07)	0.402	0.08	0.02	(0.04,0.13)	4.36×10^{-5}
	Moderate amblyopia	0.07	0.05	(-0.04,0.19)	0.344	0.12	0.05	(0.00,0.24)	0.048
	Severe amblyopia	0.10	0.03	(0.03,0.18)	0.003	0.06	0.03	(-0.02,0.14)	0.168
CNVA (Jaeger)	Mild amblyopia	0.67	0.38	(-0.26,1.59)	0.248	0.89	0.37	(0.00,1.78)	0.051
	Moderate amblyopia	0.71	0.49	(-0.47,1.88)	0.442	1.18	0.49	(-0.01,2.36)	0.052
	Severe amblyopia	0.89	0.37	(0.24,1.54)	0.003	0.39	0.39	(-0.41,1.19)	0.724
Mean Sensitivity (dB)	Mild amblyopia	-1.15	0.76	(-2.98,0.68)	0.389	-2.38	0.76	(-4.22,-0.53)	0.007
	Moderate amblyopia	-1.57	0.99	(-3.97,0.83)	0.346	-0.94	0.98	(-3.31,1.42)	1.000
	Severe amblyopia	-2.12	1.00	(-4.54,0.31)	0.108	-0.91	1.12	(-3.61,1.80)	1.000
Mean Defect (dB)	Mild amblyopia	1.16	0.74	(-0.62,2.94)	0.351	2.11	0.73	(0.33,3.88)	0.014
	Moderate amblyopia	1.68	0.99	(-0.70,4.07)	0.269	1.59	0.94	(-0.68,3.87)	0.275
	Severe amblyopia	1.96	0.86	(-0.11,4.02)	0.071	0.78	1.06	(-1.78,3.34)	1.000
Stereo Acuity (log arcsecond)	Mild amblyopia	0.02	0.15	(-0.35,0.39)	1.000	0.31	0.14	(-0.02,0.64)	0.078
	Moderate amblyopia	0.04	0.22	(-0.50,0.57)	1.000	0.09	0.26	(-0.55,0.72)	1.000
	Severe amblyopia	0.00	0.18	(-0.44,0.44)	1.000	0.48	0.18	(0.04,0.92)	0.029

Bonferroni post-hoc analyses identified different temporal windows for clinical response in different groups.
CDVA, corrected distance visual acuity; CI, confidence interval; CNVA, corrected near visual acuity; logMAR, logarithm of the minimum angle of resolution; MD, mean difference; SE, standard error.

Supplementary Table S4. Longitudinal changes in neuroimaging characteristics in the MRI subcohort

Characterisitic	Baseline (n = 17)	6-month follow-up (n = 17)	Conhe n's d	P value	P_{FDR} value
Total intracranial volume (cm³)*	1367.75 ± 91.90	1387.28 ± 93.43	0.89	0.001	0.013
Mean cortical thickness (mm)	2.80 ± 0.11	2.79 ± 0.10	-0.13	0.421	0.561
The GM volume (cm³)	769.86 ± 53.46	767.09 ± 54.97	-0.26	0.297	0.481
The WM volume (cm³)*	403.23 ± 40.57	408.31 ± 40.05	1.22	0.003	0.003
The WM					
FA	0.41 ± 0.02	0.42 ± 0.01	0.27	0.108	0.287
ADC (× 10³ mm²/s)	0.81 ± 0.03	0.81 ± 0.02	-0.25	0.319	0.481
The LGN volume (mm³)					
Right	94.66 ± 16.85	99.83 ± 21.20	0.31	0.224	0.448
Left†	88.47 ± 15.55	95.24 ± 16.22	0.58	0.029	0.139
Right optic radiations					
FA	0.43 ± 0.02	0.43 ± 0.03	-0.06	0.478	0.603
ADC (× 10³ mm²/s)	0.82 ± 0.05	0.83 ± 0.06	0.07	0.897	0.897
Left optic radiations					
FA	0.43 ± 0.02	0.43 ± 0.03	-0.05	0.298	0.481
ADC (× 10³ mm²/s)	0.87 ± 0.07	0.87 ± 0.06	-0.18	0.776	0.887
Right primary visual cortex					
Thickness (mm)	1.87 ± 0.17	1.84 ± 0.14	-0.36	0.152	0.332
Volume (mm³)	4967.88 ± 990.41	4955.41 ± 915.74	-0.05	0.831	0.890
Left primary visual cortex					
Thickness (mm)	1.74 ± 0.12	1.73 ± 0.12	-0.12	0.622	0.747
Volume (mm³)	4084.06 ± 735.38	4072.65 ± 725.24	-0.05	0.853	0.890
Right secondary visual cortex					
Thickness (mm)†	2.29 ± 0.16	2.27 ± 0.16	-0.53	0.046	0.183
Volume (mm³)	13011.88 ± 2009.07	12832.94 ± 1884.43	-0.38	0.138	0.332
Left secondary visual cortex					
Thickness (mm)*	2.26 ± 0.15	2.22 ± 0.14	-0.77	0.006	0.042
Volume (mm³)*	13006.82 ± 1904.66	12709.65 ± 1684.70	-0.75	0.007	0.042
Right middle Temporal visual area					
Thickness (mm)	2.69 ± 0.16	2.64 ± 0.13	-0.42	0.100	0.287
Volume (mm³)	4626.88 ± 851.47	4528.82 ± 756.65	-0.45	0.085	0.287
Left middle Temporal visual area					
Thickness (mm)	2.74 ± 0.10	2.73 ± 0.12	-0.24	0.328	0.481
Volume (mm³)	4277.47 ± 583.73	4243.65 ± 563.76	-0.24	0.341	0.481

Data were presented as mean ± SD, with statistical comparisons evaluated via a paired t-test.

† indicates a nominal association at an unadjusted $P < 0.05$; * Denotes significant inter-group differences.

ADC, apparent diffusion coefficient; FA, fractional anisotropy; FDR = false discovery rate; LGN, lateral geniculate nucleus; MRI, magnetic resonance imaging; WM, white matter

Supplementary Table S5. Interhemispheric comparison of posterior visual pathway morphometric and microstructural measures at the 6-month follow-up

Characterisitic	Right Hemisphere (n = 17)	Left Hemisphere (n = 17)	Cohens 'd	P value	P_{FDR} value
The LGN volume (mm^3)	99.83 ± 21.20	95.24 ± 16.22	0.23	0.349	0.393
Optic radiations					
FA	0.43 ± 0.03	0.43 ± 0.03	0.25	0.088	0.133
ADC (x 10^{-3} mm^2/s)*	0.83 ± 0.06	0.87 ± 0.06	-1.42	2.399 ×10^{-5}	1.079 ×10^{-3}
Primary visual cortex					
Thickness (mm)*	1.84 ± 0.14	1.73 ± 0.12	0.92	0.002	0.005
Volume (mm^3)*	4955.41 ± 915.74	4072.65 ± 725.24	1.77	1.733 ×10^{-6}	1.559 ×10^{-6}
Secondary visual cortex					
Thickness (mm)*	2.27 ± 0.16	2.22 ± 0.14	0.92	0.002	0.005
Volume (mm^3)	12832.94 ± 1884.43	12709.65 ± 1684.70	0.14	0.562	0.562
MT					
Thickness (mm)*	2.64 ± 0.13	2.73 ± 0.12	-0.77	0.014	0.032
Volume (mm^3)	4528.82 ± 756.65	4243.65 ± 563.76	0.36	0.154	0.199

Data are presented as mean ± SD, with statistical comparisons evaluated via a paired t-test.

* Denotes significant inter-group differences.

ADC = apparent diffusion coefficient; FA = fractional anisotropy; FDR = false discovery rate; LGN = lateral geniculate nucleus; MRI = magnetic resonance imaging; MT = Middle Temporal visual area

Supplementary Table S6. **Factors associated with improvements in mean sensitivity and corrected distance visual acuity**

Reference categories	Parameter	β	95%CI	Weight (%)	P value	P_{FDR} value
The mild amblyopia group	Postoperative time (yrs)	-0.51	(-1.21,0.18)	5.30	0.142	-
	Central lens opacity diameter (mm)*	1.00	(0.19,1.82)	18.75	0.017	-
	Corrected distant visual acuity (LogMAR)*	3.36	(1.37,5.35)	56.35	0.001	-
	The moderate amblyopia group	-1.61	(-3.28,0.06)	11.08	0.058	0.087
	The severe amblyopia group	-2.22	(-4.42,-0.01)	8.53	0.049	0.087
The moderate amblyopia group	Postoperative time (yrs)	-0.51	(-1.21,0.18)	5.47	0.142	-
	Central lens opacity diameter (mm)*	1.00	(0.19,1.82)	21.91	0.017	-
	Corrected distant visual acuity (LogMAR)*	3.36	(1.37,5.35)	56.24	0.001	-
	The mild amblyopia group	1.61	(-0.06,3.28)	13.45	0.565	0.116
	The severe amblyopia group	-0.61	(-2.72,1.51)	2.93	0.142	0.565
The severe amblyopia group	Postoperative time (yrs)	-0.51	(-1.21,0.18)	5.28	0.142	-
	Central lens opacity diameter (mm)*	1.00	(0.19,1.82)	19.66	0.017	-
	Corrected distant visual acuity (LogMAR)*	3.36	(1.37,5.35)	57.39	0.001	-
	The mild amblyopia group	2.22	(0.01,4.42)	12.82	0.049	0.098
	The moderate amblyopia group	0.61	(-1.51,2.72)	4.85	0.565	0.565

Data were analysed using multivariable linear regression. Post-hoc pairwise comparisons among the three amblyopia severity groups (mild, moderate, and severe) regarding their associations with mean sensitivity improvement were evaluated, with P values adjusted using the false discovery rate (FDR) method.

* Indicates a statistically significant association with MS improvement.

CI, confidence interval; FDR, false discovery rate; logMAR, logarithm of the minimum angle of resolution; MD, mean difference.

Supplementary Table S7. The associations between changes in neuroimaging indices and clinical visual outcomes

Supplementary Table 7a. The partial correlation analysis between changes in neuroimaging indices and corrected distance visual acuity

Neuroimaging changes	Changes in covariates	r	95%CI	P value	P_{FDR} value
The GM volume (cm³)	Total intracranial volume (cm³)	0.14	(-0.40, 0.61)	0.616	0.982
The WM volume (cm³)†	Total intracranial volume (cm³)	0.56	(0.07, 0.83)	0.029	0.358
R-LGN volume (mm³)	Total intracranial volume (cm³)	0.33	(-0.21, 0.72)	0.223	0.804
L-LGN volume (mm³)	Total intracranial volume (cm³)	-0.13	(-0.60, 0.41)	0.637	0.982
R-OR FA†	FA in the WM	-0.54	(-0.83, -0.05)	0.035	0.358
L-OR FA†	FA in the WM	-0.71	(-0.90, -0.32)	0.003	0.171
R-OR ADC (× 10³ mm²/s)	ADC in the WM (× 10³ mm²/s)	-0.07	(-0.56, 0.46)	0.802	0.982
L-OR ADC (× 10³ mm²/s)	ADC in the WM (× 10³ mm²/s)	-0.19	(-0.64, 0.36)	0.499	0.982
R-V1 volume (mm³)	Total intracranial volume (cm³)	-0.31	(-0.71, 0.24)	0.265	0.820
L-V1 volume (mm³)	Total intracranial volume (cm³)	-0.13	(-0.60,0.41)	0.653	0.982
R-V2 volume (mm³)	Total intracranial volume (cm³)	-0.06	(-0.56, 0.47)	0.832	0.982
L-V2 volume (mm³)	Total intracranial volume (cm³)	0.29	(-0.26, 0.70)	0.287	0.820
R-MT volume (mm³)	Total intracranial volume (cm³)	0.45	(-0.08, 0.78)	0.091	0.547
L-MT volume (mm³)	Total intracranial volume (cm³)	-0.05	(-0.55, 0.48)	0.867	0.982
R-V1 thickness (mm)	Mean cortical thickness (mm)	0.04	(-0.49, 0.54)	0.899	0.982
L-V1 thickness (mm)	Mean cortical thickness (mm)	-0.18	(-0.63, 0.37)	0.521	0.975
R-V2 thickness (mm)	Mean cortical thickness (mm)	-0.17	(-0.63, 0.37)	0.536	0.975
L-V2 thickness (mm)	Mean cortical thickness (mm)	0.05	(-0.47,0.55)	0.856	0.982
R-MT thickness (mm)	Mean cortical thickness (mm)	0.03	(-0.49, 0.53)	0.926	0.982
L-MT thickness (mm)	Mean cortical thickness (mm)	-0.38	(-0.75,0.16)	0.164	0.783

† indicates a nominal association at an unadjusted $P < 0.05$; none of the associations remained statistically significant after false discovery rate correction.

ADC, apparent diffusion coefficient; CI, confidence interval; FA, fractional anisotropy; FDR = false discovery rate; L, left; LGN, lateral geniculate nucleus; MT, middle temporal visual area; OR, optic radiation; R, right; V1, primary visual cortex; V2, secondary visual cortex; WM, white matter

Supplementary Table S7b. The partial correlation analysis between changes in neuroimaging indices and mean defect

Neuroimaging changes	Changes in covariates	r	95%CI	P value	P_{FDR} value
The GM volume (cm^3)	Total intracranial volume (cm^3)	-0.25	(-0.68, 0.30)	0.363	0.900
The WM volume (cm^3)	Total intracranial volume (cm^3)	0.38	(-0.16, 0.75)	0.158	0.783
R-LGN volume (mm^3)	Total intracranial volume (cm^3)	0.25	(-0.30, 0.68)	0.371	0.900
L-LGN volume (mm^3)†	Total intracranial volume (cm^3)	-0.54	(-0.83, -0.05)	0.036	0.358
R-OR FA	FA in the WM	0.02	(-0.49, 0.53)	0.932	0.982
L-OR FA	FA in the WM	0.07	(-0.46, 0.56)	0.806	0.982
R-OR ADC ($\times 10^3$ mm^2/s)	ADC in the WM ($\times 10^3$ mm^2/s)	0.13	(-0.41, 0.60)	0.637	0.982
L-OR ADC ($\times 10^3$ mm^2/s)	ADC in the WM ($\times 10^3$ mm^2/s)	0.00	(-0.51, 0.51)	0.995	0.998
R-V1 volume (mm^3)	Total intracranial volume (cm^3)	0.02	(-0.49, 0.53)	0.933	0.982
L-V1 volume (mm^3)	Total intracranial volume (cm^3)	-0.32	(-0.72, 0.23)	0.243	0.811
R-V2 volume (mm^3)	Total intracranial volume (cm^3)	-0.37	(-0.74, 0.17)	0.170	0.783
L-V2 volume (mm^3)	Total intracranial volume (cm^3)	0.09	(-0.44, 0.58)	0.738	0.982
R-MT volume (mm^3)	Total intracranial volume (cm^3)	0.10	(-0.43, 0.59)	0.710	0.982
L-MT volume (mm^3)	Total intracranial volume (cm^3)	-0.33	(-0.72, 0.22)	0.228	0.804
R-V1 thickness (mm)	Mean cortical thickness (mm)	-0.03	(-0.53, 0.49)	0.923	0.982
L-V1 thickness (mm)	Mean cortical thickness (mm)	-0.18	(-0.63, 0.36)	0.517	0.975
R-V2 thickness (mm)	Mean cortical thickness (mm)	-0.26	(-0.68, 0.29)	0.342	0.900
L-V2 thickness (mm)	Mean cortical thickness (mm)	-0.18	(-0.63, 0.37)	0.527	0.975
R-MT thickness (mm)	Mean cortical thickness (mm)	-0.25	(-0.67, 0.30)	0.375	0.900
L-MT thickness (mm)†	Mean cortical thickness (mm)	-0.56	(-0.83, -0.06)	0.031	0.358

† indicates a nominal association at an unadjusted $P < 0.05$; none of the associations remained statistically significant after false discovery rate correction.

ADC, apparent diffusion coefficient; CI, confidence interval; FA, fractional anisotropy; FDR = false discovery rate; L, left; LGN, lateral geniculate nucleus; MT, middle temporal visual area; OR, optic radiation; R, right; V1, primary visual cortex; V2, secondary visual cortex; WM, white matter

Supplementary Table S7c. The partial correlation analysis between changes in neuroimaging indices and mean sensitivity

Neuroimaging changes	Changes in covariates	r	95%CI	P value	P_{FDR} value
The GM volume (cm^3)	Total intracranial volume (cm^3)	-0.03	(-0.53, 0.49)	0.929	0.982
The WM volume (cm^3)	Total intracranial volume (cm^3)	-0.50	(-0.80, 0.02)	0.060	0.452
R-LGN volume (mm^3)	Total intracranial volume (cm^3)	-0.35	(-0.73, 0.20)	0.202	0.804
L-LGN volume (mm^3)	Total intracranial volume (cm^3)	0.46	(-0.07, 0.79)	0.087	0.547
R-OR FA	FA in the WM	0.12	(-0.42, 0.60)	0.672	0.982
L-OR FA	FA in the WM	0.10	(-0.44, 0.58)	0.732	0.982
R-OR ADC ($\times 10^3$ mm^2/s)	ADC in the WM ($\times 10^3$ mm^2/s)	-0.13	(-0.13, 0.65)	0.655	0.982
L-OR ADC ($\times 10^3$ mm^2/s)	ADC in the WM ($\times 10^3$ mm^2/s)	0.04	(-0.48, 0.54)	0.896	0.982
R-V1 volume (mm^3)	Total intracranial volume (cm^3)	0.00	(-0.51, 0.51)	0.998	0.998
L-V1 volume (mm^3)	Total intracranial volume (cm^3)	0.14	(-0.40, 0.61)	0.622	0.982
R-V2 volume (mm^3)	Total intracranial volume (cm^3)	0.33	(-0.22, 0.72)	0.226	0.804
L-V2 volume (mm^3)	Total intracranial volume (cm^3)	-0.21	(-0.65, 0.33)	0.443	0.975
R-MT volume (mm^3)	Total intracranial volume (cm^3)	-0.30	(-0.70, 0.25)	0.275	0.820
L-MT volume (mm^3)	Total intracranial volume (cm^3)	0.50	(-0.01, 0.81)	0.057	0.452
R-V1 thickness (mm)	Mean cortical thickness (mm)	-0.01	(-0.52, 0.51)	0.984	0.998
L-V1 thickness (mm)	Mean cortical thickness (mm)	0.15	(-0.39, 0.62)	0.594	0.982
R-V2 thickness (mm)	Mean cortical thickness (mm)	0.18	(-0.37, 0.63)	0.532	0.975
L-V2 thickness (mm)	Mean cortical thickness (mm)	0.18	(-0.36, 0.64)	0.512	0.975
R-MT thickness (mm)	Mean cortical thickness (mm)	0.02	(-0.48, 0.53)	0.931	0.982
L-MT thickness (mm)†	Mean cortical thickness (mm)	0.64	(0.19, 0.87)	0.010	0.358

† indicates a nominal association at an unadjusted $P < 0.05$; none of the associations remained statistically significant after false discovery rate correction.

ADC, apparent diffusion coefficient; CI, confidence interval; FA, fractional anisotropy; FDR = false discovery rate; L, left; LGN, lateral geniculate nucleus; MT, middle temporal visual area; OR, optic radiation; R, right; V1, primary visual cortex; V2, secondary visual cortex; WM, white matter

Supplementary Table S8. Therapeutic patient satisfaction

On a scale of 0 (not likely at all) to 10 (extremely likely), how likely is it that you would recommend the combined therapeutics to someone else with a lazy eye?	Total group (N=53)[1]
0	0 (0%)
1	0 (0%)
2	1 (1.89%)
3	0 (0%)
4	2 (3.77%)
5	1 (1.89%)
6	1 (1.89%)
7	5 (5.66%)
8	3 (9.43%)
9	9 (16.98%)
10	31 (58.49%)
**NPS Calculation**	
% responses 9 or 10	75.47%
% responses 0 to 6	9.43%
NPS Score	66.04
**How likely would you be to choose the combined therapeutics over eye-patching to treat your child's amblyopia in the future?[2]**	**Total group (N=27)[2]**
Very unlikely	0 (0%)
unlikely	1 (3.70%)
Neutral	3 (11.11%)
likely	5 (18.52%)
Very likely	18 (66.67%)

Data presented as: n (%).

[1]Based on participants with available data at baseline and 6-month visits.

[2]Based on participants with available data at baseline and 6-month visits, and with prior patching history.

Supplementary Table S9. The repeatability, reliability, and variability of central visual field examinations at each visit

Supplementary Table 9a. Test-retest reliability of central visual field indices (mean deviation and mean sensitivity) across study visits

Visit	Parameters	N	Single meaurement		Averaged measurement	
			ICC	95%CI	ICC	95%CI
Baseline	Mean deviation (dB)	55	0.95	(0.93,0.97)	0.98	(0.97,0.98)
	Mean sensitivity (dB)	55	0.92	(0.88,0.95)	0.96	(0.94,0.97)
month 3	Mean deviation (dB)	51	0.96	(0.94,0.97)	0.98	(0.97,0.99)
	Mean sensitivity (dB)	51	0.93	(0.89,0.95)	0.96	(0.94,0.98)
month 6	Mean deviation (dB)	53	0.98	(0.96,0.99)	0.99	(0.98,0.99)
	Mean sensitivity (dB)	53	0.95	(0.92,0.97)	0.97	(0.96,0.99)

Intraclass correlation coefficients (ICCs) were evaluated using a two-way mixed-effects model (absolute agreement).

CI, confidence interval; ICC, intraclass correlation coefficient; N, number of participants at visit.

Supplementary Table S9b. Measurement agreement of central visual field indices (mean deviation and mean sensitivity) across study visits

Visit	Parameters	N	Median difference	95%LoA	CR
Baseline	Mean deviation (dB)	55	0.6	(-2.12,1.86)	1.93
	Mean sensitivity (dB)	55	-0.8	(-2.40,2.52)	2.40
month 3	Mean deviation (dB)	51	0.4	(-1.60,1.84)	1.64
	Mean sensitivity (dB)	51	-0.7	(-2.11,2.15)	2.02
month 6	Mean deviation (dB)	53	-0.5	(-1.93,1.07)	1.73
	Mean sensitivity (dB)	53	0.7	(-1.67,2.10)	1.83

Measurement agreement was evaluated using non-parametric Bland-Altman analysis.

CR, coefficient of repeatability; dB, decibel; LoA, limit of agreement; N, number of participants at visit.
